# Analysis of Clinical Characteristics and Risk Factors of Plastic Bronchitis in Children With *Mycoplasma pneumoniae* Pneumonia

**DOI:** 10.3389/fped.2021.735093

**Published:** 2021-10-18

**Authors:** Haiqin Zhong, Rong Yin, Ran Zhao, Kun Jiang, Chao Sun, Xiaoyan Dong

**Affiliations:** Department of Respiratory Medicine, Shanghai Children's Hospital, Shanghai Jiao Tong University, Shanghai, China

**Keywords:** *Mycoplasma pneumoniae* pneumonia, plastic bronchitis, children, risk factors, clinical characteristics

## Abstract

**Objective:** To analyze the clinical characteristics of plastic bronchitis (PB) in children with *Mycoplasma pneumoniae* pneumonia (MPP) in order to explore its risk factors.

**Methods:** A retrospective analysis was performed in MPP children receiving bronchoscopy admitted to department of respiratory medicine in Shanghai Children's Hospital from January 2018 to December 2020. According to the bronchoscopic findings, the patients were divided into PB group and non-PB group. The clinical manifestations, laboratory examination, etiology, treatment methods and outcomes of the children were analyzed. Logistic regression was used to analyze the risk factors for PB in children with MPP.

**Results:** A total of 296 children with MPP were enrolled in the study, including 42 (14.2%) children in the PB group and 254 (85.8%) children in the non-PB group. There was no difference in the ratios of gender, age, proportion of fever, cough, wet rales, and wheezing rales between the two groups (*P* > 0.05). The univariate analysis showed that there were significant differences between the PB group and the non-PB group in LDH, D-dimer, CD3+CD4+(%), CD3+CD4+/CD3+CD8+, CD3 count, CD4 count, CD8 count, complement 3, IL8, IL-1β, IL-2, IL-10 (*P* < 0.05). The multivariate logistic regression analysis showed that fever duration > 12 d, IL-8 > 2,721.33 pg/ml, LDH > 482 U/L and complement 3 <1.02 g/L were independent risk factors for PB in children with MPP.

**Conclusions:** Children with PB caused by MPP have protracted fever, a strong inflammatory response and immune function disturbance.

## Introduction

*Mycoplasma pneumoniae* (MP) is a common pathogen of community-acquired pneumonia (CAP) in children. *Mycoplasma pneumoniae* pneumonia (MPP) accounts for 10–40% of CAP in hospitalized children (1–3). Plastic bronchitis (PB) refers to a disease in which endogenetic foreign bodies locally or extensively block the bronchus, leading to partial or complete ventilation dysfunction of the lung (4). Clinically, the disease onset and progression are rapid, with the manifestations of a high fever, cough, progressive dyspnea and difficulty improving hypoxemia, which can be quickly relieved after plastic is removed by bronchoscopy (5). In plastic bronchitis caused by infection, common pathogens include bacteria (such as *Streptococcus pneumoniae*) and viruses (such as influenza virus and adenovirus) (6, 7). However, recently, the role of MP in PB has gradually received increased attention. In this study, 296 children with MPP and underwent bronchoscopy were retrospectively analyzed to explore the risk factors of PB in children with MPP.

## Methods

### Patients and Definitions

Children with MPP who were admitted to department of respiratory medicine in Shanghai Children's Hospital from January 2018 to December 2020 and treated with bronchoscopy were selected as subjects. The patients were divided into PB group and non-PB group according to whether there was plastic shape under bronchoscope. The inclusion criteria were as follows: ① The condition met the diagnostic criteria for *Mycoplasma pneumoniae* pneumonia (8) i.e., CAP cases met one of the following criteria: (i) a single dose of serum *Mycoplasma pneumoniae* antibody MP-Ab ≥ 1:160; (ii) the titer of MP-Ab increased or decreased by 4 times or more in the recovery and acute stages; (iii) the copy number of MP-DNA in nasopharyngeal swabs, sputum, bronchoalveolar lavage fluid (BALF) and pleural effusion was >500/ml. ② The condition met the diagnostic criteria of plastic bronchitis: taken out plastic foreign body by bronchoscopy. ③ Informed consent was signed for bronchoscopy examination. ④ Complete hospitalization data were available. The exclusion criteria were as follows: ① previous recurrent respiratory tract infection, asthma, chronic lung disease, severe blood system disease or immune deficiency disease; ② foreign body inhalation; ③ incomplete hospitalization data.

### Etiological Detection

After admission, all the children were subjected to the following: *Mycoplasma pneumoniae* antibody identification, detection of MP-DNA using a nasopharyngeal swab, detection of blood respiratory pathogen IgM antibody nine (eosinophilic lung *Legionella* bacteria, *Mycoplasma pneumoniae, Chlamydia, Rickettsia*, adenovirus pneumonia, syncytial virus, influenza A virus, influenza B virus, and parainfluenza), detection of phlegm respiratory pathogen eight (influenza A and influenza B, respiratory syncytial virus, adenovirus, metapneumovirus, parainfluenza virus type 1, 2, 3), tuberculosis antibody detection, sputum culture, blood culture, and alveolar lavage fluid bacterial culture identification.

### Data Collection

The clinical data of children with MPP were collected and mainly included the following: (1) general information: name, sex, age, admission time, discharge time, length of stay, and hospitalization cost; (2) clinical manifestations: fever, cough, shortness of breath, wheezing and other symptoms, lung rales, wheezing and other signs; (3) laboratory tests: routine blood tests, inflammatory markers, blood biochemistry, humoral immunity, cellular immunity, etiology, and BALF cytokines; (4) imaging examination results; (5) treatment and clinical outcome.

### Statistical Analysis

SPSS 24.0 statistical software was used for descriptive analysis of the data. Enumeration data were expressed as percentages (%), measurement data with a normal distribution were described as means ± standard deviation, and measurement data with a non-normal distribution were described as medians. The chi-square test was used for categorical data, and the *t*-test and Mann-Whitney U test were used for continuous data. Logistic regression analysis was used to examine risk factors that were significant in the univariate analysis. *P* < 0.05 was considered statistically significant.

## Results

### General Information

A total of 296 MPP children were eligible for inclusion. The PB group accounted for 42 (14.2%) cases and non-PB group for 254 (85.8%) cases. The mean age of PB and non-PB patients was 6.21 ± 3.00 years and 6.42 ± 2.60 years, respectively. Male percentage was 45.2% (19 of 42) in the PB patients and 48.0% (122 of 254) in the non-PB patients. There were no significant differences in gender composition and average age between the two groups (*P* > 0.05).

### Clinical Features

Varying degrees of fever were found for 40 (95.2%) patients with PB and 227 (89.4%) patients without PB. The average durations of fever for the PB group and non-PB group were 9.55 ± 5.19 d and 7.79 ± 4.24 d, respectively. The duration of fever of the PB group were longer than that of the non-PB group (*P* < 0.05).

All patients had cough. Thirty-three patients (78.6%) had wet rales, and 9 patients with PB (21.4%) had a wheezing sound. One hundred seventy-eight patients (70.1%) had wet rales, and 48 patients (18.9%) without PB had a wheezing sound. There were no significant differences in wet rales and wheezing rales between the two groups (Table 1).

**Table 1:** Clinical features and laboratory values between the PB group and the non-PB group in children with MPP.

**Variables**	**PB group**	**Non-PB group**	***P***
Male to female ratio	19/23	122/132	0.737
Age in years	6.21 ± 3.00	6.42 ± 2.60	0.656
Length of hospital stay, days	12.00 (7.00, 15.25)	7.00 (6.00, 9.00)	<0.001^*^
Fever duration	9.55 ± 5.19	7.79 ± 4.24	0.017^*^
Wet rales	33 (78.6%)	178 (70.1%)	0.260
Wheezing rales	9 (21.4%)	48 (18.9%)	0.700
WBC (×10^9^/L)	6.64 (4.71, 8.77)	7.82 ± 3.71	0.233
*N* (%)	62.19 ± 19.55	60.53 ± 14.05	0.505
PCT (ng/mL)	0.38 (0.18, 0.93)	0.39 (0.28, 1.37)	0.292
ESR (mm/h)	52.05 ± 29.06	50.32 ± 23.77	0.472
CRP (mg/L)	30.00 (14.00, 54.00)	23.00 (10.00, 49.00)	0.576
LDH (U/L)	464.00 (352.00, 634.50)	334.00 (283.50, 392.00)	<0.001^*^
Ferritin (ng/mL)	253.30 (109.68, 557.15)	253.90 (146.1, 739.10)	0.468
Fibrinogen (g/L)	3.86 ± 1.14	3.78 ± 0.81	0.593
D-dimer (mg/L FEU)	1.21 (0.77, 2.64)	0.58 (0.39, 1.03)	<0.001^*^
CD3+ (%)	64.26 ± 8.16	66.40 ± 8.18	0.132
CD3+CD4+ (%)	31.79 ± 7.72	35.70 ± 7.15	0.002^*^
CD3+CD8 (%)	27.50 ± 7.85	25.51 ± 6.23	0.076
CD3+CD4+/ CD3+CD8+	1.19 (0.83, 1.57)	1.45 (1.10, 1.79)	0.004^*^
CD3-CD19 (%)	21.29 (16.49, 31.32)	19.55 (16.48, 29.00)	0.520
CD3-CD16+56+ (%)	9.88 ± 5.42	10.38 ± 5.59	0.615
CD3+ count	1.00 (0.51, 1.41)	1.38 (1.01, 2.02)	<0.001^*^
CD4+ count	0.51 (0.25, 0.71)	0.73 (0.52, 1.05)	<0.001^*^
CD8+ count	0.35 (0.24, 0.56)	0.51 (0.36, 0.75)	0.001^*^
IgA (g/L)	1.21 (0.85, 2.02)	1.17 (0.78,1.72)	0.250
IgG (g/L)	9.34 (7.71, 10.68)	9.49 (7.81, 11.00)	0.715
IgM (g/L)	1.29 (1.09, 1.88)	1.37 (1.07, 1.81)	0.963
IgE (IU/mL)	125.00 (48.98, 324.75)	110.00 (39.90, 292.00)	0.328
Complement 3 (g/L)	1.11 ± 0.23	1.21 ± 0.19	0.026^*^
Complement 4 (g/L)	0.28 ± 0.10	0.32 ± 0.11	0.127
**Cytokines in BALF**			
IL-8 (pg/ml)	1,532.75 (787.41, 3,482.62)	669.83 (185.00, 1,400.43)	<0.001^*^
IL-1β (pg/ml)	188.62 (12.16, 809.41)	44.33 (0.10, 249.47)	0.002^*^
IL-2 (pg/ml)	0.10 (0.10, 0.10)	0.10 (0.10, 0.10)	0.043^*^
IL-10 (pg/ml)	0.10 (0.10, 0.10)	0.10 (0.10, 0.10)	0.003^*^
IL-6 (pg/ml)	0.10 (0.10, 73.03)	0.10 (0.10, 0.10)	0.064

* *P < 0.05*.

### Laboratory Tests

There was no significant difference in the white blood cell count (WBC), neutrophil (N%), C-reactive protein (CRP), procalcitonin (PCT), erythrocyte sedimentation rate (ESR), Ferritin, Fibrinogen between the two groups, but lactate dehydrogenase (LDH), D-dimer levels in children with PB were significantly higher than those in non-PB group, as shown in Table 1. The levels of CD3+CD4+(%), CD3+CD4+/CD3+CD8+, CD3+ count, CD4+ count and CD8+ count in the T-lymphocyte subsets in the PB group and the non-PB group was statistically significant. There were no significant differences in the levels of IgA, IgG, IgM, and IgE in humoral immunity between the two groups. The level of Complement 3 (C3) in the PB group was lower than that in the non-PB group. Significant differences (*P* < 0.05) were demonstrated in IL8, IL-1β, IL-2, IL-10 of BALF between the two groups (Table 1).

### Etiological Examination

The mixed infection rate in PB group was higher than that in non-PB group [57.1% (24/42 cases) vs. 39.0% (99/254 cases)]. Among the 42 cases of PB, 6 cases (14.3%; 3 cases of Streptococcus pneumoniae, 2 cases of Klebsiella pneumoniae, and 1 case of Acinetobacter baumannii) were combined with bacterial infection. Fourteen cases (33.3%; including 5 cases of adenovirus, 3 cases of influenza B, 3 cases of rhinovirus, 2 cases of respiratory syncytial virus, and 1 case of parainfluenza) were combined with virus infection. Four cases (9.5%) were infected with multiple pathogens. In the non-PB group, the detection rate of mixed bacterial infection was 8.3% (21/254), among which streptococcus pneumoniae had the highest detection rate of 5.5% (14/254). The detection of mixed virus infection was 34.3% (87/254) and rhinovirus had the highest rate of 9.1% (23/254).

### Radiographic Examination

All the children had undergone chest X-ray or CT examination after admission, and pulmonary inflammation was found in all of them. There were 18 cases (42.9%) of pleural effusion in the PB group, and 28 cases (11.0%) in the non-PB group. The proportion of pleural effusion in the PB group was higher than that in the non-PB group (χ^2^ = 27.826, *P* < 0.05). The proportion of atelectasis in the two groups was 11.9% (5/42) and 4.7% (12/254), respectively. There was no significant difference (χ^2^ = 3.433, *P* > 0.05).

### Clinical Treatment

All patients were administered intravenous broad-spectrum antibiotics after admission. They had undergone bronchoscopy and alveolar lavage, during which different degrees of congestion and edema were observed in the bronchial mucosa, showing inflammatory changes of the endometrium, accompanied by many yellow and white mucus and phlegm plugs to block the trachea, and the suction tube was yellow with white branches. Twenty-five patients received one bronchoscopy and alveolar lavage, and 17 patients received more than two bronchoscopies and alveolar lavages in the PB group. The proportion of patients needing multiple bronchoscopy therapy (≥2 times) in the PB group was significantly higher than that in the non-PB group [17(40.5%) vs. 24 (9.5%), χ^2^ = 29.076, *P* < 0.05)]. After alveolar lavage treatment, the clinical manifestations were improved to varying degrees. The average hospital stays of the PB group and non-PB group were 12.0 and 7.0 d, respectively. The length of hospital stays in PB group was longer than that in non-PB group (*P* < 0.05). All patients were discharged from the hospital after treatment.

### Risk Factors for PB Caused by MPP

#### The Univariate Analysis

The univariate analysis showed that there were significant differences between the PB group and the non-PB group in length of hospital stay, fever duration, LDH, D-dimer, CD3+CD4+(%), CD3+CD4+/CD3+CD8+, CD3+ count, CD4+ count, CD8+ count, complement 3 (C3), IL8, IL-1β, IL-2, IL-10 (*P* < 0.05), as shown in Table 1.

#### The Multivariate Logistic Regression Analysis

Whether PB occurred or not was taken as the response variable. In univariate analysis, 14 variables of statistically significant length of hospital stay, fever duration, LDH, D-dimer, CD3+CD4+(%), CD3+CD4+/ CD3+CD8+, CD3+ count, CD4+ count, CD8+ count, C3, IL8, IL-1β, IL-2 and IL-10 were independent variables. The measurement data were converted into dichotomous variables according to the upper or lower quartile of each independent factor for further Logistic regression analysis. The results showed that fever duration > 12 d, IL-8 > 2,721.33 pg/ml, LDH > 482 U/L and C3 < 1.02 g/L were independent risk factors for PB in children with MPP, as shown in Table 2.

**Table 2:** Risk factors for PB in children with MPP.

**Independent variables**	**B**	**S.E**.	**Waldχ^2^**	***P***	**OR**	**95% CI**
Fever duration > 12 d	1.537	0.731	4.415	0.036	4.650	1.109~19.499
IL-8 > 2,721.33 pg/ml	1.607	0.790	4.142	0.042	4.990	1.061~23.462
LDH > 482 U/L	1.637	0.812	4.059	0.044	5.138	1.045~25.252
C3 < 1.02 g/L	1.722	0.807	4.550	0.033	5.595	1.159~27.227

## Discussion

PB is a relatively rare respiratory disease with different clinical symptoms and a potentially fatal risk. Its onset is insidious, and its diagnosis is mainly based on bronchoscopy and pathologic examination of plastic foreign bodies. The common diseases of childhood PB are lower respiratory tract infection, cyanotic congenital heart disease, bronchial asthma and respiratory allergic diseases, as well as sickle cell anemia, globin dysplasia anemia, cystic fibrosis, and primary nephrotic syndrome (9, 10). In recent years, the clinical manifestations of MPP in children have varied, and the cases of severe MPP and refractory MPP have significantly increased, while few clinical reports of secondary PB have been published (11).

The present study showed that the characteristics of onset age were related to the higher incidence of MPP in school-aged children. School-aged children with mature immune systems are prone to strong immune responses, leading to airway mucosal damage that easily forms mucus plugs to block the airway (12). Cai et al. (13) found that MP is the first pathogenic bacterium detected in children with PB after pneumonia, suggesting that MP infection is the primary pathogenic bacterium causing PB. The clinical manifestations of MPP combined with PB mainly include fever and cough, with persistent high fever and a long heat course. Some patients exhibit wheezing and dyspnea, and the degree of dyspnea is determined by the size and location of pathogenic bacteria and the bronchial plastic model. The main signs are rapid breathing, three concave signs, and decreased respiratory sounds on the affected side. In the present study, the children had a long heat course, averaging 12 days. Long-term fever indicated that inflammation is not effectively controlled. Inflammatory stimulation caused tracheal mucosa injury, necrosis, increased mucus secretion, and accelerated the formation of plastic objects.

CRP is an acute response protein synthesized by the liver that can be detected 12–48 h after acute infection. The CRP level is correlated with the severity of infection. The results showed there was no significant difference in the CRP levels between the PB and non-PB groups of children with MPP. It was not consistent with Hua's (14) report. LDH is a cytoplasmic enzyme in many organs. An increased LDH level can reflect the degree of tissue damage and lysis. Lee et al. (15) found that the CRP and LDH levels can be used to reflect the therapeutic effect of MPP in children. Wang et al. (16) showed that age, the total heat course, and the CRP, LDH and ESR levels in children with MPP mucus plug blockage were higher than those in the healthy subject group, suggesting that the increase in CRP, LDH and ESR can be used as an indicator of airway mucus embolism in children with MPP.

D-dimer and fibrinogen are commonly used clinical indicators of coagulation and fibrinolysis systems, respectively. D-dimer is a sensitive indicator reflecting the body's hypercoagulability and thrombosis. Increased D-dimer levels can increase microthrombosis at the lesion site, resulting in necrosis of microvessels and tissue cells, necrosis and abscesses of fibrous epithelial cells, and increased mucus gland secretion of mucus thrombi in the airway. Literatures showed that the increased D-dimer and fibrinogen levels in patients with severe pneumonia can be used to evaluate the severity of severe pneumonia (17, 18). Han et al. (19) showed that the levels of D-dimer and fibrinogen in the PB group were higher than those in the non-PB group, suggesting that the degree of coagulation dysfunction and inflammatory damage in the PB group was more serious.

Humoral immunity and cellular immunity are involved in the pathogenesis of MP infection. In this study, CD3+CD4+(%), CD3+CD4+/CD3+CD8+, CD3+ count, CD4+ count, CD8+ count and C3 in the PB group were lower than that in the non-PB group. It suggested that there was stronger immune response in the PB group, which was related to the formation of plastic. The levels of the BALF cytokines IL-8 and IL-1β were increased in most MPP patients with PB, suggesting that overexpression of the inflammatory cytokines IL-8 and IL-1β may be involved in the pathological process of PB in MPP. IL-8 is a chemokine produced by macrophages and other cells, such as epithelial cells, airway smooth muscle cells, and endothelial cells (20). IL-1β is mainly produced by activated innate immune cells such as macrophages and monocytes (21). Gui et al. (22) found that the levels of IL-8 and IL-1β in the BALF of children with MPP were significantly higher than those in the BALF of children in the bacterial and control groups, suggesting that the high expression levels of IL-8 and IL-1β in BALF are involved in the occurrence and development of MPP and play important roles in MPP exacerbation. Cai et al. (23) found that the levels of IL-8, IL-1β, and IL-6 in BALF in the SMPP group were higher than those in the non-SMPP group, suggesting that the level of cytokine secretion was increased because of local immune damage in the lung tissue after infection. Therefore, we speculate that MP infection lead to increased release of inflammatory mediators, inflammatory luminal stenosis, increased capillary permeability, and increased gland secretion, leading to the occurrence of PB.

As MP damages airway mucosal cells and mucociliary clearance system, meanwhile, the body's immunity is decreased after MP infection, often resulting in mixed infection. The present study showed that the mixed infection rate of children with MPP combined with PB was as high as 52.4%, mixed viruses were the most common, and adenovirus was the most common virus infection. Zhang et al. (24) found that human rhinovirus, bocavirus and respiratory syncytial virus are the main infections of mixed viruses in MPP-hospitalized children. Guo et al. (25) found that plastic bronchitis of MPP is prone to virus infection, with EB virus and influenza B being common. Wei et al. (26) found that children with influenza complicated with PB are prone to mixed bacterial infection, mostly *Streptococcus pneumoniae*. CAP mixed infection in children is more likely to lead to PB.

Three main mechanisms are associated with PB: airway inflammation causes substantial mucus secretion in the airway; airway inflammation leads to necrosis and shedding of respiratory epithelium, mucosa edema, and decreased respiratory tract cleaning ability; lymphatic leakage occurs in the airway. The pathological mechanism of PB caused by MP infection includes the following: MP infection directly adheres to the airway epithelium and causes direct damage to the airway epithelium through cytotoxic action or causes damage through an immune mechanism. The results in this study showed that fever duration > 12 d, IL-8 > 2,721.33 pg/ml, LDH > 482 U/L and C3 < 1.02 g/L were independent risk factors for PB in children with MPP. Zhang et al. (27) showed that when MPP children were aged ≥ 3 years, the heat course was ≥10 days, the CRP level was ≥40 mg/L, and the LDH level was ≥350 U/L, the formation of a mucus thrombus in the airway is possible. Shi et al. (28) found that in MPP children aged ≥ 6 years, a D-dimer level ≥ 0.9 mg/L and the presence of atelectasis on imaging are independent risk factors for the formation of airway mucus thrombus in MPP children.

Early improvement of alveolar ventilation is the key to the treatment of MPP combined with PB, and the removal of plastic secretions via bronchoscopy is the only effective method to treat PB (29). All the children in the present study received complete bronchoscopy after admission, and part of them received 2 or more bronchoscopies and alveolar lavages, indicating that only one alveolar lavage treatment may not completely remove plastic substances. After bronchoscopy and alveolar lavage, the clinical manifestations and influence were improved significantly in all children. After bronchoscopy and alveolar lavage treatment for contact airway obstruction, all children were quickly removed from the device. Further support for bronchoscopy is the main treatment method to quickly relieve the clinical symptoms of PB.

The present study has some shortcomings. As a single-center retrospective study, this study lacks pathological analysis of bronchial plastic patterns, and there may be deviation in the selection of data. Therefore, multicenter and large-sample data are required to further explore the pathogenesis and intervention measures of PB caused by MPP, and follow-up should be conducted.

In summary, Children with PB caused by MPP have protracted fever, a strong inflammatory response and immune function disturbance. Fever duration > 12 d, IL-8 > 2,721.33 pg/ml, LDH > 482 U/L and C3 < 1.02 g/L were independent risk factors for PB in children with MPP. Timely bronchoscopy and alveolar lavage treatment can obtain a good therapeutic effect to prevent the occurrence of sequelae.

## Data Availability Statement

The original contributions generated for the study are included in the article/supplementary material, further inquiries can be directed to the corresponding author/s.

## Ethics Statement

The studies involving human participants were reviewed and approved by The Ethics Committee of Shanghai Children's Hospital. Written informed consent to participate in this study was provided by the participants' legal guardian/next of kin.

## Author Contributions

HZ summarized the data and wrote the paper. RY collected the patient's information. RZ modified the paper. KJ and CS operated the bronchoscope. XD was a major contributor in revising the manuscript. All authors read and approved the final manuscript.

## Funding

This study was supported by the funding from Subject of Shanghai Administration of Traditional Chinese Medicine (Project Number: ZHYY-ZXYJHZX-201920) and subproject of STAR program of Shanghai Jiao Tong University (Project Number: 20190102).

## Conflict of Interest

The authors declare that the research was conducted in the absence of any commercial or financial relationships that could be construed as a potential conflict of interest.

## Publisher's Note

All claims expressed in this article are solely those of the authors and do not necessarily represent those of their affiliated organizations, or those of the publisher, the editors and the reviewers. Any product that may be evaluated in this article, or claim that may be made by its manufacturer, is not guaranteed or endorsed by the publisher.
